# Lactopeptine

**Published:** 1878-06

**Authors:** 


*The Canada Medical Record* bears record to the value of "Lactopeptine," in the following extract: "This is a remedy which we have prescribed during the last four months with a good deal of satisfaction to ourselves and benefit to our patients. It certainly is a very valuable preparation for various forms of indigestion, and is composed of pepsin, pancreatine, diastare, ptyalin, lactic and hydro-chloric acids, and we invite our readers to give it a trial."

